# Modular Fuel Cell Stacks Using 3D-Printed Resin-Based Frames

**DOI:** 10.1021/acsomega.6c07546

**Published:** 2026-10-26

**Authors:** Iñaki Requena-Leal, Miguel García-López, Justo Lobato, Manuel A. Rodrigo

**Affiliations:** Department of Chemical Engineering. Faculty of Chemical Sciences & Technologies, University of Castilla—La Mancha, Campus Universitario s/n 13071 Ciudad Real, Spain

## Abstract

This study presents the design, fabrication, and performance evaluation of modular fuel cell stacks using 3D-printed resin-based frames, targeting portable and decentralized energy storage applications. The innovative design prioritizes low cost, mechanical robustness, and ease of component replacement, making it suitable for deployment in harsh environments such as agricultural settings. Unlike conventional graphite-based bipolar plates, the resin structures offer printability and modularity, enabling rapid prototyping and field-level maintenance. Electrochemical characterization of single cells and stacks demonstrated high reproducibility, with optimal current densities of 150–165 mA·cm^–2^ and peak power densities above 55 mW·cm^–2^ at 20 °C. Series and parallel configurations of up to five cells were tested, revealing that stack performance is highly sensitive to the quality of the membrane electrode assemblies (MEAs). Variability in MEA electrocatalytic properties led to significant differences in polarization resistance and power output, underscoring the importance of component uniformity. Series connections increased voltage, while parallel configurations enhanced current density, although both reached similar maximum power densities. Current densities at which the maximums are obtained are very different, being around 20–30 mA·cm^–2^ for the series connection and 80–100 mA·cm^–2^ for the parallel connection. The stack of 5 MEAs reached up to 6.05 W while the stack of 3 MEAs reached 4.15 W. Operational tests under realistic conditions (using air as the oxidant and hydrogen from pressurized canisters) confirmed the system’s robustness. During the test, the stack of three MEAs was sequentially supplied with the gas contained in five 1-L canisters pressurized at 4 bar and it maintained a stable 0.5 A output over 3.29 h, with a hydrogen utilization efficiency of 0.6 Wh·mg^–1^ H_2_. These results are obtained in the frame of a project about smart irrigation of crops and values obtained have validated the feasibility of 3D-printed fuel cell stacks for integration into these renewable energy systems, particularly for irrigation in water-scarce regions. Compared to conventional batteries, this approach offers a promising scalable, cost-effective, and field-serviceable alternative for sustainable energy storage.

## Graphical abstract

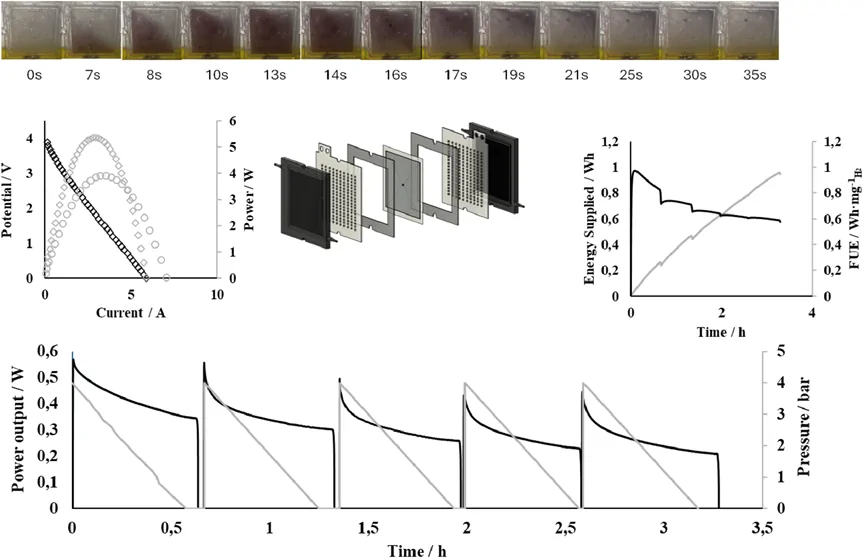

## 1 Introduction

The conversion of the chemical energy stored in the hydrogen molecule into electrical energy through electrochemical fuel cell technologies has been under development for several years in a stepwise manner.^1–3^ At different historical moments, significant advances have been made in specific technologies, leading to the current prominence of two main types: solid oxide electrolyte technology for high-temperature applications,^4,5^ and polymer electrolyte membrane (PEM) technology for operation under milder conditions.^6,7^ The latter has reached a very high level of technological readiness,^8,9^ with numerous equipment suppliers offering solutions across a wide range of power requirements. Unfortunately, it is not the electrochemical reaction itself but rather the technological integration that represents the most sensitive aspect of the technology.^10,11^ In commercial systems, water management and heat exchange become critically important factors.^12–15^

For this reason, commercially available equipment is increasingly becoming a ’black box’ for users, with significant difficulties in being repaired by third parties in the event of malfunction or failure.^16,17^ This trend runs counter to the emerging culture of the circular economy, which is being promoted to enhance the sustainability of our planet.^18,19^ In this context, the development of systems that can be more easily implemented without requiring specialized knowledge is becoming a necessity. One tool that is significantly contributing to the democratization of knowledge is 3D printing, which enables the fabrication of components, even at the household level.^20–22^

One possible future scenario involves the commercialization of systems that, although less efficient than commercially optimized “black box” solutions, could be easily repaired. It is important to consider that the use of fuel cell technologies is increasingly linked to renewable energy sources, particularly in terms of aligning energy production and consumption curves.^23,24^ In this regard, ease of use and maintenance are beginning to outweigh the benefits of highly integrated systems, especially when such integration results in complex maintenance or repair procedures.^25^

The widespread adoption of 3D printing is becoming an important ally, as it enables the fabrication of essential components for equipment at the domestic level and can serve as a significant driver for improving sustainability by allowing simple and rapid repairs.^26–28^ This represents a major cultural shift, but it is also crucial to begin implementing changes in this direction by developing technologies that extend the lifespan of equipment and reduce dependence on suppliers.^29,30^

Within this framework, the potential sustainability of additively manufactured fuel-cell systems should be considered not only in terms of material selection, but also in terms of repairability, component replacement, and distributed manufacturing. Modular architectures may extend equipment lifetime by allowing damaged components to be individually replaced rather than requiring disposal of the complete stack. In addition, additive manufacturing can reduce fabrication waste and enable the local production of spare parts, potentially decreasing transportation requirements and dependence on specialized suppliers.

In this context, the objective pursued with this work is to develop a resin structure to generate the frames that will form fuel cells for portable applications that can be integrated into small-sized energy storage systems. The required specifications are low weight, low cost, high mechanical strength, and easy replacement. Instead of using the more conventional graphite frames, it has been preferred to sacrifice the conductivity of the frame and its function as a bipolar plate for a system that can be printed in resin by a domestic printer and thus can be easily implemented in domestic, industrial or even agricultural environments, where extreme weather conditions and the working environment are excessively aggressive for the materials normally used in this technology.^31^ In this type of systems, the speed of part replacement is of extreme importance, and avoiding dependence on a critical industrial supplier is essential for realistic operability. The stack of cells is prepared to be operated at ambient or moderate temperatures (not exceeding 80 °C, considering the thermal resistance of the resin used), and the outlet of each frame is connected to a device that allows the collection of the water that forms and condenses, preventing it from reaching the next frame when the cells are stacked to form a stack.

The cell will be characterized both in standalone operation mode and when assembled in stacks, through the evaluation of polarization and power curves under various conditions aimed at clarifying its robustness. This characterization will conclude with a usage study in a scenario where the cell is consecutively fed by five hydrogen canisters, emulating the second phase of an energy regulation process based on energy storage, in which electrical energy is regenerated from hydrogen previously produced during a stage that utilizes surplus energy to generate this chemical species.

## 2 Materials and Methods

### 2.1 Fuel Cell Frames

The cell frames have been designed with an inlet and an outlet pipe and can be used interchangeably to introduce fuel (typically hydrogen) or the oxidant, being possible to use not only with air or oxygen but also with chlorine, as the material is resistant to it. This is of vital importance when aiming to develop novel fuel cell technologies such as the chlor-alkali type, where the chemical resistance of construction materials becomes a critical factor for their advancement. The flow arrangement is in a checkerboard pattern and between every two frames there is an electrode membrane assembly. Figure 1 shows a schematic of the cell configuration, where the structure developed as a frame and its arrangement with respect to the membrane-electrode assemblies to form a stack of cells can be observed. Every two units can accommodate a membrane-electrode assembly (MEA) with an active area of 36 cm^2^, which receives electrical current through perforated stainless steel current feeders. These connectors are designed for easy integration with systems that consume electricity.

**Figure 1:**
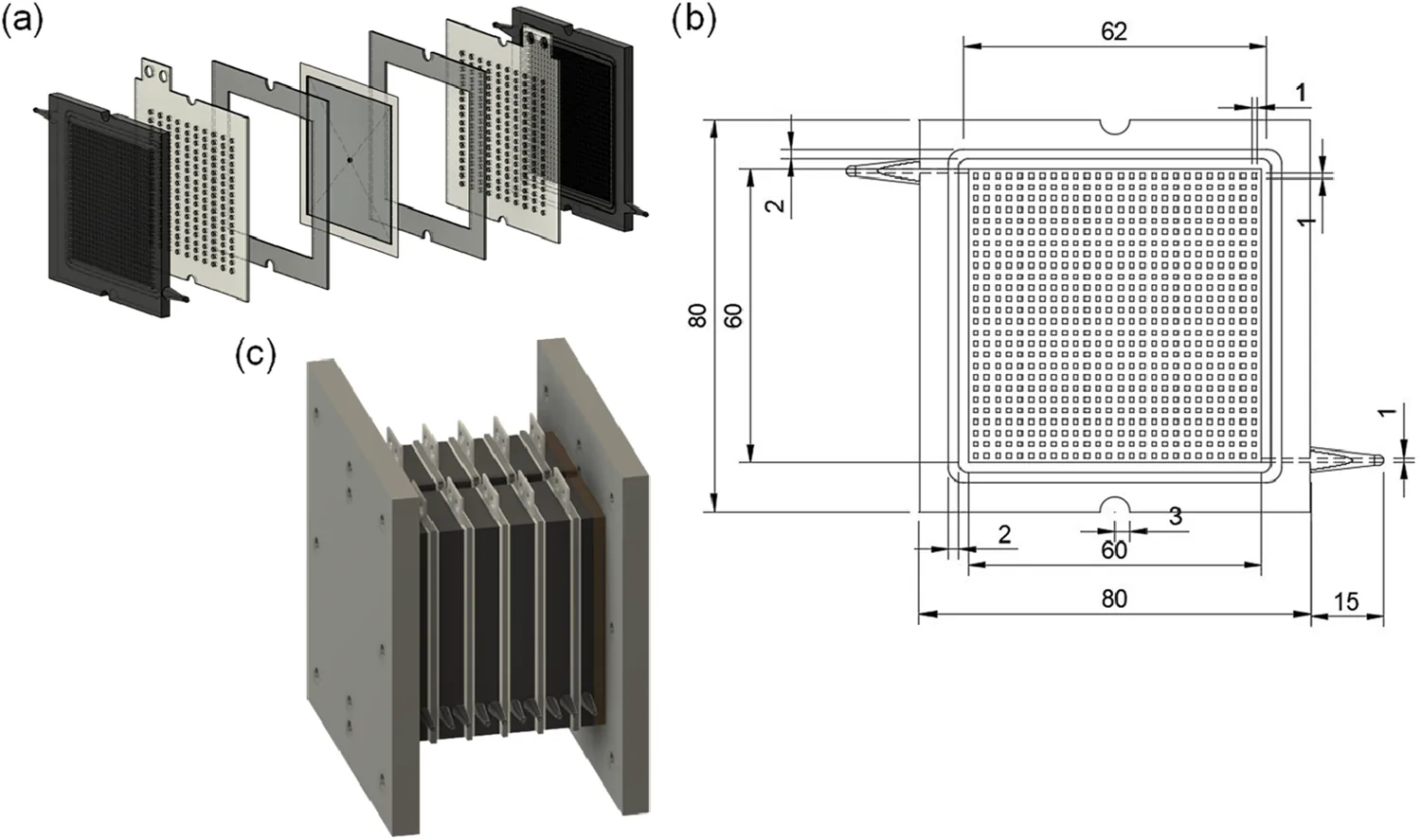
(a) Layout of fuel cells made with 3-D printing technology. (b) detail of the frame of the fuel cell (c) stack with five cells.

Additionally, the structure that conducts the flow of fluids through it can be seen. It was designed to achieve an effective distribution of reactants, enabling a high degree of uniformity in the current density distribution across the electrode surfaces, an essential factor for ensuring good cell performance in terms of energy conversion and durability. Besides the cell frames, the installation of gaskets is necessary to ensure the sealing of each compartment, and the ends of the stacks are equipped with two robust plates, also made of resin, to allow the bolted closure of the entire structure by applying sufficient torque to ensure the system’s proper sealing.

### 2.2 Experimental System

The characterization studies of the performance of individual fuel cells and the corresponding stacks were conducted using a simple laboratory setup. Each cell was connected to a hydrogen cylinder (200 bar, 99.999% purity, AirLiquide) and an oxygen cylinder (200 bar, 99.995%, Carburos Metálicos), as well as to a potentiostat (Metrohm Autolab PGSTAT 302N with 20A Booster). To assess performance differences with various oxidants, in some experiments the oxygen cylinder was replaced with compressed air supplied by the compressor installed in the laboratory. For polarization and power curve measurements, the current was fixed, and the voltage was recorded once stabilized. For the Electrochemical impedance spectroscopy (EIS) studies, the applied voltage depended on the device configuration (0.87 V for single cells and stacks connected in parallel; 2 V for the three single cells connected in series; and 2,5 V for five single cells connected in series). The amplitude was set to 0.05 V, and the frequency range was established between 0.1 and 10,000 Hz, using the FRA module of the potentiostat. Depending on the experiment, the cells were fed with hydrogen at a flow rate of either 115 mL·min^–1^ or 230 mL·min^–1^. The air and oxygen flow rates were 200 mL·min^–1^. Five different stack configurations were tested, consisting of 2, 3, or 5 MEAs connected using various electrical connection schemes.

The development of these stacks of cells is part of projects aimed at creating chlor-alkali electrolytic technology for use in automotive applications and, especially in agricultural irrigation, powered by hydrogen generated using solar energy. This hydrogen is stored in cylinders (or canisters on a smaller scale) during production and subsequently fed from these canisters into the cell stack (in this study a 3-MEAs stack with series connection was used). For this reason, to evaluate their efficiency under the intended operating conditions, during the operational test, five 1-L pressurized canisters, each charged with hydrogen at 4 bar, were used. The stored hydrogen was consecutively fed into the stack, with the objective of maintaining a constant current of 0.5 A while measuring the resulting voltage. The hydrogen feed rate to the stack was 115 mL·min^–1^, and air was used as the oxidant, considering that the use of high-purity oxygen is economically unfeasible for the intended applications. The air flow rate introduced was 300 mL·min^–1^.

## 3 Results and Discussion

### 3.1 Fluid Dynamic Characterization of the Reactors

To evaluate the fluid dynamics of the fuel cell, several tests were conducted in which the frames were installed in mockups to track tracers, and the frames were fed with both gaseous and liquid streams. Thus, to evaluate the behavior with gases, a tracer consisting of hot gas was introduced stepwise into the cell, and thermographic monitoring of the temperature distribution at different feed rates (within the operating range considered in the frame design) was performed to determine the transient process until reaching the new steady-state situation where the temperature distribution became uniform again.

As shown in Figure 2, the fluid initially distributes in the lower positions, from where it advances diagonally to the outlet, reaching a stabilized response situation with respect to the input step after about 8 min. Considering that the response of a first-order dynamic system to a step change (eq 1) stabilizes (response range above 98%) after about 3–4 times τ, this means that the frame generates a dynamic delay of about 2–2.5 min, which is a very suitable value. There does not seem to be a significant influence of the response speed concerning the introduced tracer gas (high temperature) flow rate

1 $$y\left(t\right)=\mathrm{ka}\left(1-e^{-t/\tau }\right)$$

This type of behavior was confirmed when the cell was operated with liquids instead of gases, introducing a solution of EBT dye at a concentration of 0.02 g L^–1^as a tracer for this confirmation. As shown in part b of the Figure, the same color distribution was observed in the cell as that recorded with temperature when the gaseous tracer was introduced. Additionally, experiments were conducted by introducing saline tracers (3 M NaCl solutions) as impulse disturbances. The results obtained from this latter experiment (part c of the Figure 2) were used to determine the mean residence time and the dimensionless Peclet number, whose values are presented in the table included in the inset of the Figure 2. The high Peclet numbers indicate a significant contribution of convective mass transport relative to diffusive transport in this cell, which is a desired feature in the design and confirms its success from a fluid dynamic perspective.

**Figure 2:**
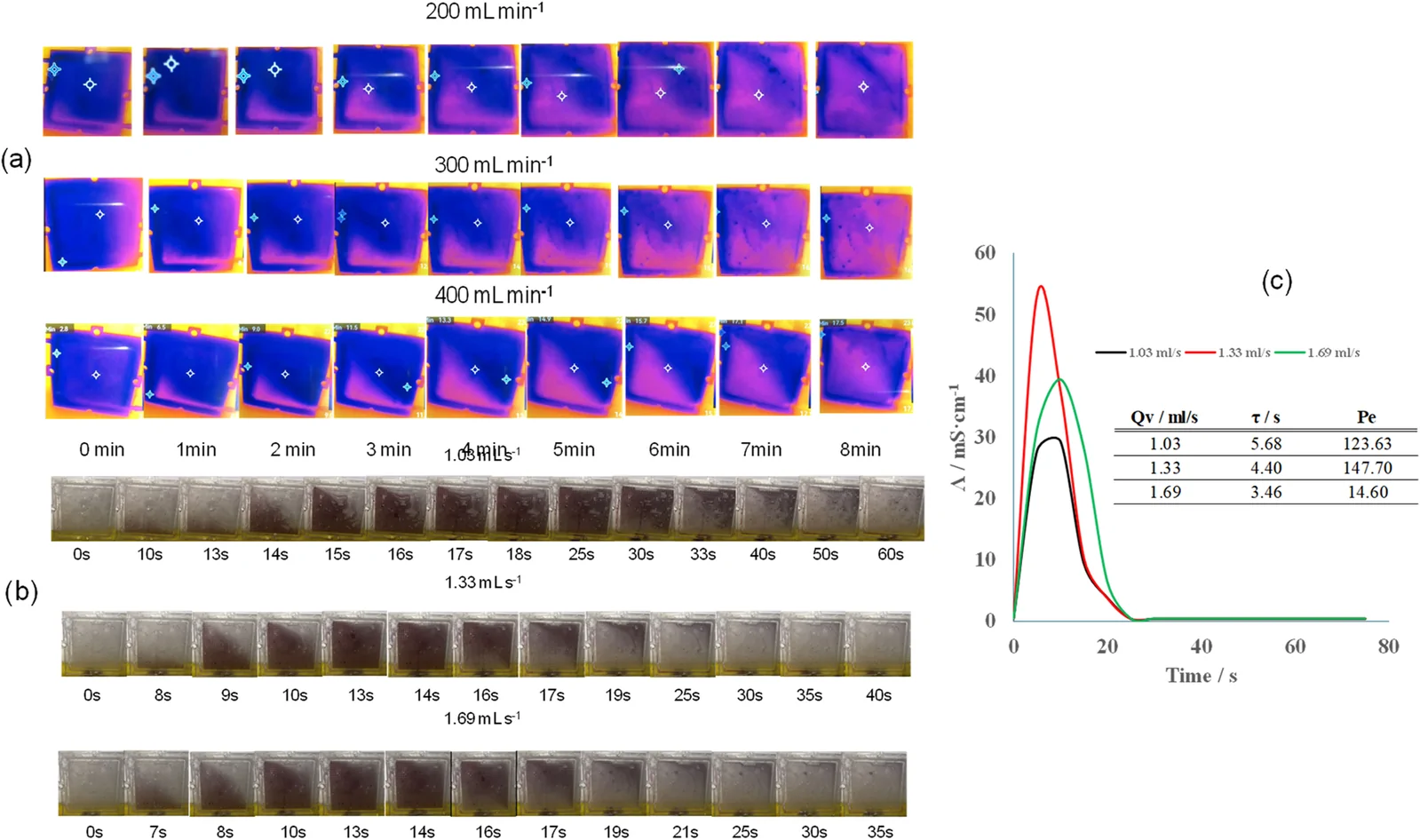
Fluid dynamic characterization of the frames with different tracer tests. (a) Carrier: Cold Air 600 mL min^–1^ Tracer: Hot air; (b) carrier: Tap water. Tracer: 3.0 M NaCl brackish solution; (c) carrier: Tap water. Tracer: Eriochrome black T 0.02 g L^–1^. Panel (c): Black line: 1.03 mL·s^–1^; Red Line: 1.33 mL·s^–1^; Green Line: 1.69 mL·s^–1^.

### 3.2 Performance of the Fuel Cell

Figure 3 shows the behavior of three simple cells equipped with different membrane electrode assemblies that have been produced in the same experimental series, with the aim of determining the reproducibility of the response, as well as the main operating characteristics of the cells. In each test, different MEAs and different frames were used to form the final cell, although the manufacturing procedures were the same.

**Figure 3:**
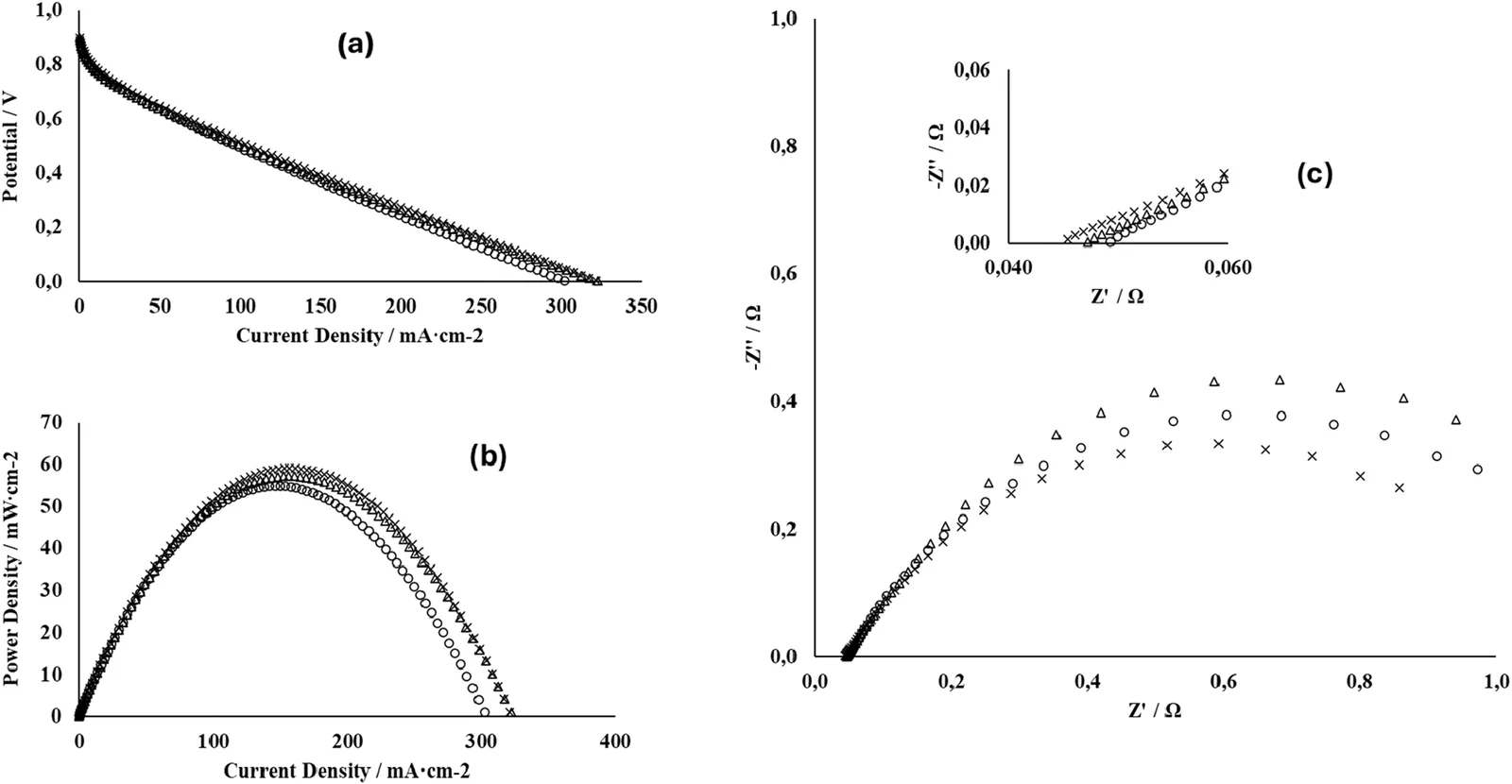
Performance reproducibility of three different fuel cells equipped with MEAS and frames made following the same procedure. Hydrogen flow rate: 200 mL·min^–1^. Oxygen flow rate: (a) Polarization curves comparison of the three main fuel cells used on the stack. (b) Power density curves of the three fuel cells used on the stack. (c) Complete Nyquist plot for each fuel cell involved on the stack. onset: zoom of the ohmic resistance region. FC1(△); FC2(○): FC3­(x).

The figure shows the polarization and power curves for the three single fuel cells are similar, indicating that the obtained system is highly reproducible, as all the points obtained in the three cells that have been operated independently fall exactly in the same area, indicating a maximum operating current density between 300 and 325 mA·cm^–2^ (10.8–11.7 A) and an optimal operating current density of around 150–165 mA·cm^–2^ (5.4–5.94 A), for which a maximum specific power slightly above 55 mW·cm^–2^ (1.98W) is obtained. Open circuit potential is near 0.9 V. These values can be considered high, considering that the operating temperature is 20 °C and the platinum load of the electrodes is 0.7 mg cm^–2^. These benchmarks were obtained feeding the system with 230 mL·min^–1^ of hydrogen and pure oxygen (99.995%) with a flow rate of 200 mL·min^–1^. The figure also shows the electrochemical impedance spectra, where once again the high reproducibility of the results achieved can be confirmed, as well as the good ionic conductivity characteristics (enlargement shown in part d). The results obtained in the polarization curves and in the EIS have been respectively fitted to the models indicated in eq 2 and to a Rs (Rp CPE) model, and the parameters obtained in all three cases are shown in Table 1. Among the data presented, the most remarkable observation is the minimal difference observed, which attests to the robustness and reproducibility of technology, as well as the low value of the ohmic contribution.

2 V=a+b·log(j)+c·j

**Table 1:** Fitting Parameters of Polarization Curves and EIS to the Proposed Mathematical Models

ID	max voltage/V	max current density/mA·cm^‑2^	max power density/mW·cm^‑2^	A V	B V dec^–1^	C Ω	*R*_s_ (mΩ)	*R*_p_ (mΩ)	CPE mMho·s^N^	*N*
FC1	0.99	327.471	57.258	0.723	0.093	0.0001	47.9	85.8	18.5	0.752
FC2	0.99	306.829	54.856	0.726	0.0931	0.0001	49.5	82.9	203	0.737
FC3	0.99	326.157	59.056	0.722	0.093	0.0001	43.5	56.7	120	0.727

### 3.3 Integration of Cells into the Stacks

Figure 4 shows the polarization and power curves and the operation of the coupling of two cells in series, in an operational test where the cell was progressively asked to operate at higher voltages in potentiostatic operation mode.

**Figure 4:**
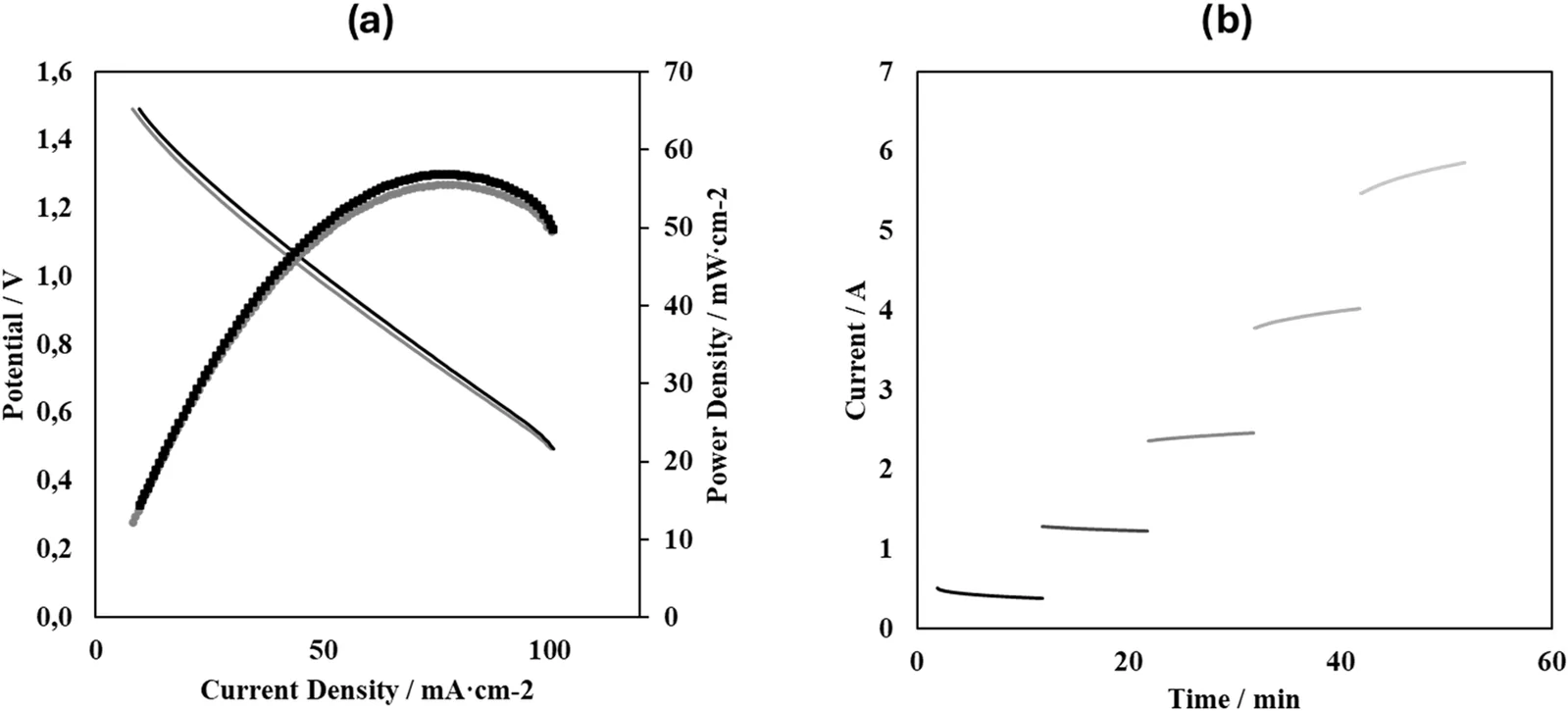
Staking of fuel cells and short operation performance tests with two MEAS with similar performance. Part (a): Polarization and power density curves for the two-cell stack at the beginning (gray lines) and after operating the cell for 60 min (black lines). Part (b): Short-term operation of the two-cell stack for its activation at: 1.5 V (black line), 1.3 V (light black line), 1.1 V (dark gray line), 0.9 V (Gray line) and 0.7 V (light gray line).

It can be observed that the response in operation is quite uniform in terms of the current intensity produced during operation, and only when the cell is asked to operate under more demanding conditions (with a high voltage), a longer stabilization time required. However, it can also be seen that there are no significant discrepancies in the polarization and power curves after the test, with the performance being slightly better after the test, which may indicate the need for system activation. Regarding the maximum power output, it is observed that the stack composed of two single cells achieves its maximum approximately at half the current density compared to the individual cells (75–80 mA·cm^–2^), while delivering twice the power (4.09 W for the coupled cells versus 2.12 W for the individual ones). This is attributed to the fact that the reaction area, and consequently the current distribution area, is doubled in the stack configuration. Therefore, this result indicates that the pairing of two individual cells using 3D-printed frames has been successful.

To continue evaluating the performance of the cell stack and the issues arising from introducing membranes with lower performance into the stack, it was decided to expand the previous stack with a new MEA that had slightly inferior operating characteristics compared to the three previously evaluated. This is a typical situation in the operation of fuel cell stacks, where the degradation of a single cell can significantly impact the overall performance of the system, becoming the limiting factor in its efficiency. The polarization and power curve of this new MEA are shown in Part a of Figure 5, where it can be observed that although it maintains the same open circuit potential (OCP) as the three previous cells, the maximum current density it can reach is one-third lower than that obtained by the three previously studied MEAs (around 200 mA·cm^–2^ compared to the more than 300 mA·cm^–2^ achieved by the previous ones, that is 7.2 A) and the optimal is slightly below 100 mA·cm^–2^ (3.6 A), being capable of delivering a maximum power of 35 mW·cm^–2^ (1.26 W). According to the electrochemical impedance spectrum (Part b), this lower performance is not associated with the electrolyte conductivity (onset) but with inferior electrocatalytic characteristics of the electrodes, as can be seen in the RCT and CPA values.

**Figure 5:**
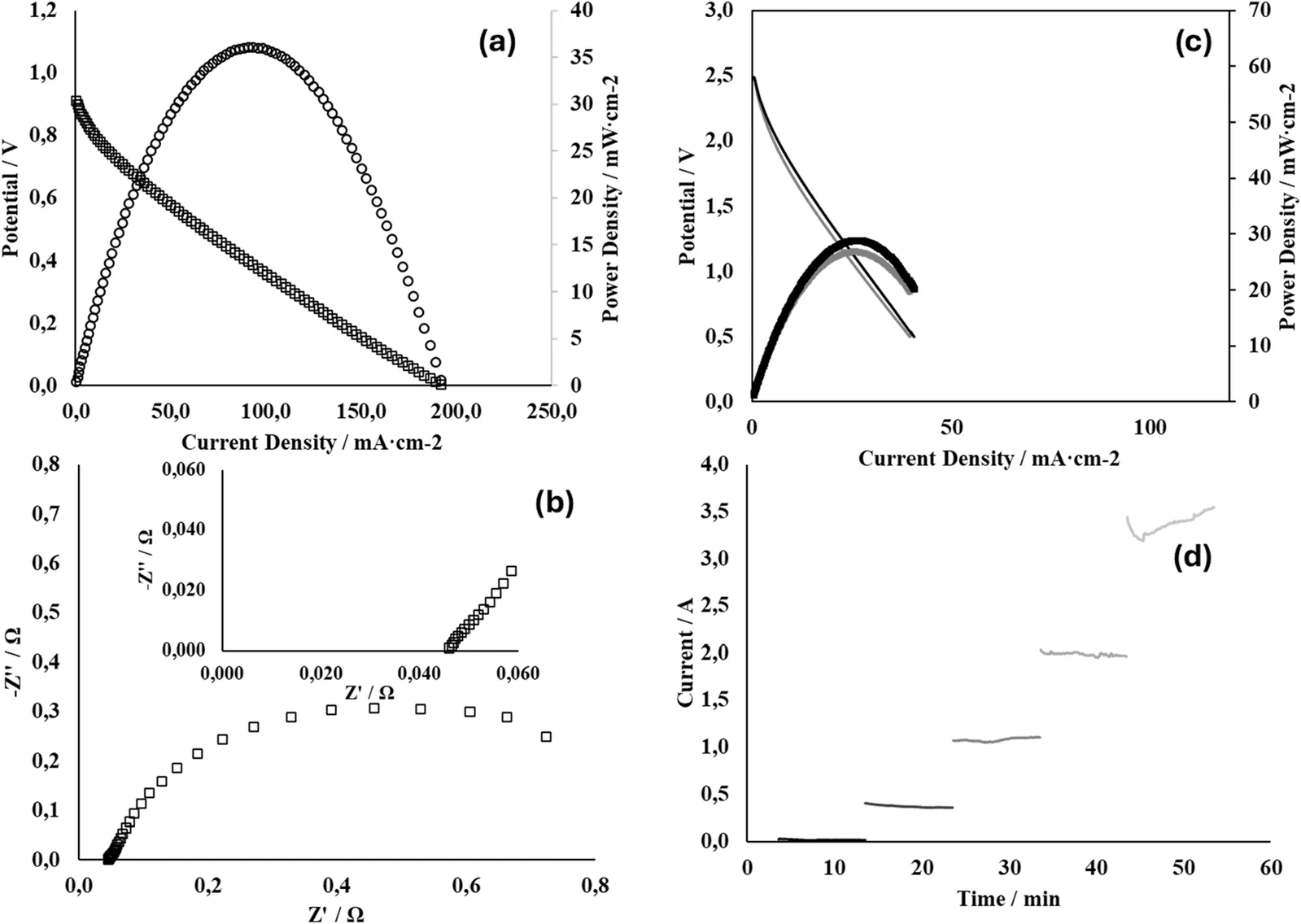
Stacking of fuel cells and short operation test with one cell with a significantly worse performance than other two. Part (a): Polarization (○) and power density (□) curves for the new cell added to the stack. Part (b): Nyquist plot of the new fuel cell added to the stack. Part (c): Polarization and Power density curves at the beginning (gray lines) and after 60 min of operation (black lines) of the fuel cell stack. Part (d): Short-term operation of the stack at different cell voltages: 2.5 V (black line), 2.1 V (light black line), 1.7 V (dark gray line), 1.3 V (Gray line) and 0.7 V (light gray line).

When coupling this cell to the stack formed by the previous two, it is observed that its lower performance significantly affects the overall performance of the stack, limiting its operation. As with the stack composed of two cells, a better response is obtained in the polarization and power curves once the system has been operating for several minutes, and it is observed that the maximum power density of the stack of 3 MEAs is limited to 28.84 mW·cm^–2^, a value much lower than the 56.84 mW cm^–2^ obtained by the stack of 2 MEAs. Consequently, the absolute power output is slightly lower than the two cell stack (3.13 W versus 4.10 W), but it is achieved at well below half the current density (41 versus 102 mA·cm^–2^), which is explained in terms of the much worse performance of the third MEA that influenced on the global performance of the stack.

Additionally, the voltage that the system can reach is much higher since the OPC of the new cell is the same as that of the previous cells. Regarding the stepwise increase in voltage performed in the operational test, it is observed that the system stabilizes very quickly, as was the case with the stack composed of two cells, and only when the system becomes more demanding (higher voltage values requested) does the response become more unstable, although in any case the response stabilizes. The current intensity values are consistent with those indicated by the polarization curve and, although they demonstrate the feasibility of stacking cells with the resin structure developed in this work, they also highlight the need to use membrane electrode assemblies of the highest possible quality and reproducibility, as the results show how an assembly with inferior performance can affect the overall operation of the stack.

From a practical implementation perspective, these findings have direct implications for stack assembly and maintenance. The results indicate that stack performance is highly dependent on MEA quality. Therefore, practical deployment should incorporate quality-control protocols, including verification of catalyst loading, membrane hydration conditions, and polarization-curve screening of individual cells before stack assembly. An additional advantage of the modular architecture developed in this work is that underperforming MEAs can be rapidly identified and replaced without requiring the replacement of the entire stack, thereby reducing downtime and maintenance costs.

### 3.4 Electrical Connection of the MEAS within the Stack

Considering the diversity of performance results that can be obtained in the operation of stacks using MEAs with different characteristics, evidenced in the previous test, and that, unfortunately, this is the most common situation faced in the real use of such devices, two coupling tests were decided to study the operation of the electrical connection in series and parallel with the cell structure developed in this work using very differentiated MEAs for stacking, whose characteristics are shown in parts a and b of Figure 6 in terms of polarization and power curves and electrochemical impedance spectrum (EIS) of the singles MEAS and in parts c and d for the stacking of three (MEAs 1, 2, and 3) and five cells (all MEAs) with electrical connection in series and parallel.

**Figure 6:**
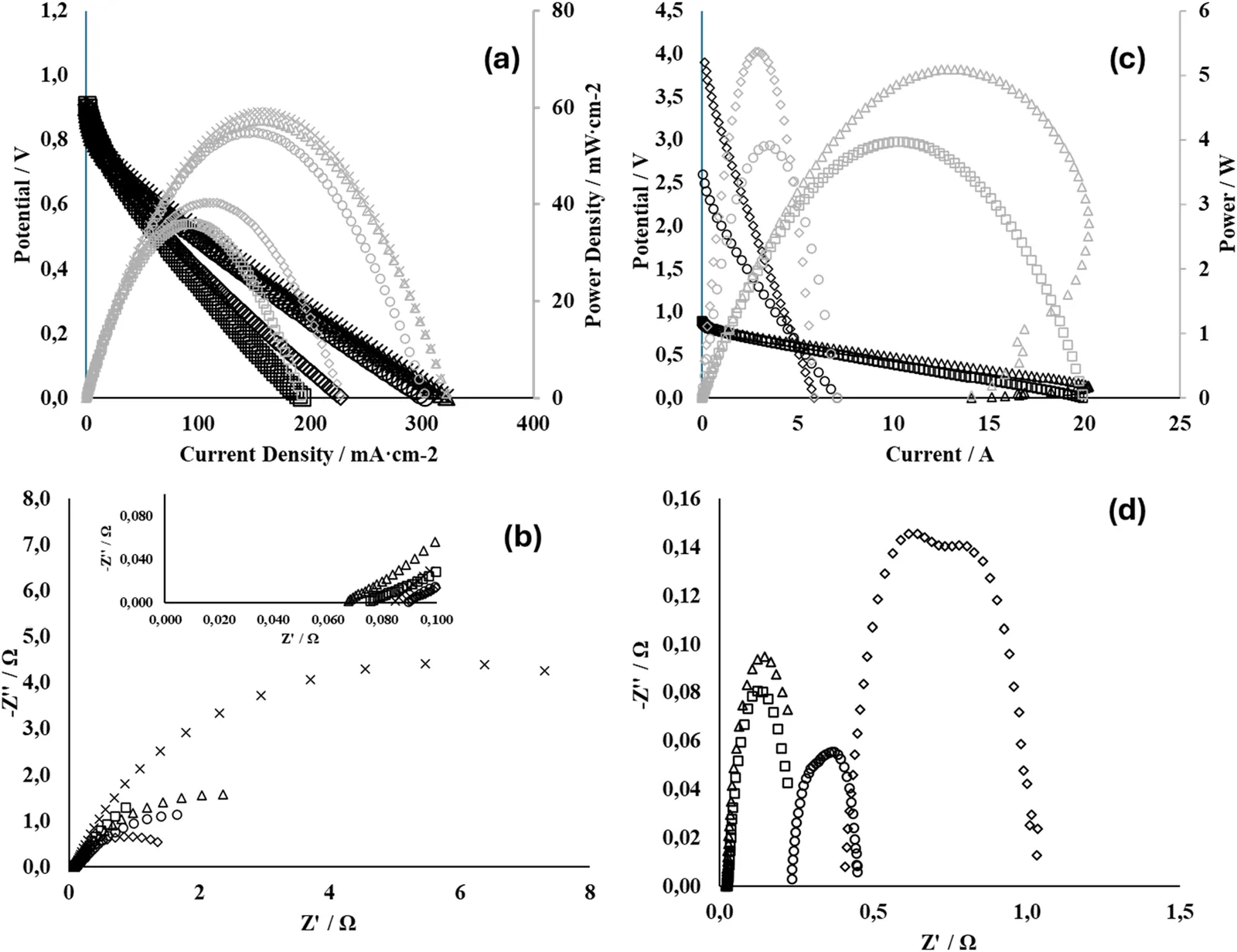
Performance of stacks of MEAS electrically connected in series and parallel. Part (a): Comparison of polarization (black symbols) and power (gray symbols) curves of all five single fuel cell (FCx) composing the stack. FC1(□), FC2(◇), FC3(△), FC4(○) and FC5­(x). Part (b): Nyquist plots for each fuel cell. FC1(□), FC2(◇), FC3(△), FC4(○) and FC5­(x). Part (c): Comparison of polarization (black symbols) and power (gray symbols) of each stack configuration. ○ (stack S3S, 3 MEAs, series connection), □ (stack S3P, 3 MEAs, parallel connection); ◇ (stack S5S, 5 MEAs, series connection); △ (stack S5P, 5 MEAs, parallel connection). Part (d): Nyquist plots for each fuel cell stack configuration. ○ (stack S3S), □ (stack S3P); ◇ (stack S5S); △ (stack S5P).

Regarding the MEAs included in the stack, their very different characteristics can be confirmed with parameters shown in Table 2 fitted from polarization curves to eq 1 and from the fitting of EIS data to the R (R CPE) model.

**Table 2:** Fitting Parameters of Polarization Curves and EIS of the MEAS Shown in Figure 6 to the Proposed Mathematical Models

ID	max voltage/V	max current density/mA·cm^‑2^	max power density/mW·cm^‑2^	A V	B V dec^–1^	C Ω	*R*_s_ (mΩ)	*R*_p_ (mΩ)	CPE mMho·s^N^	*N*
FC1	0.99	225.872	42.052	0.7452	0.0939	0.0001	74.9	82.7	205	0.72
FC2	0.99	168.271	30.476	0.7619	0.0945	0.0001	89.7	54	252	0.736
FC3	0.99	164.583	25.606	0.7631	0.0945	0.0001	83.1	54.5	50.3	0.768
FC4	0.99	122.748	19.936	0.7786	0.095	0.0001	89.7	103	44.5	0.729
FC5	0.99	122.240	18.263	0.7738	0.0949	0.0001	84.5	159	64.3	0.818

As seen, despite there were no significant discrepancies in OPC among the five MEAs, this is not the case of the performance because maximum operating current densities in the range of 120 to 225 mA·cm^–2^ and maximum power between 18 and 42 mW·cm^–2^ are obtained by the cells. According to the EIS, these differences are not related to the electrolyte conductivity but rather to the electrocatalytic properties of the MEAs, affecting both the polarization resistance (*R*_p_) and the constant phase element (CPE), with variations reaching up to a factor of 5.

Table 3 compares the fitted parameters of the curves corresponding to the four evaluated stacks.

**Table 3:** Fitting Parameters of Polarization Curves and EIS of the Stacks Shown in Figure 6 to the Proposed Mathematical Models

ID	max voltage/V	max current density/mA·cm^‑2^	max power density/mW·cm^–2^	A V	B V dec^‑1^	C Ω	*R*_s_ (mΩ)	*R*_p_ (mΩ)	CPE mMho·s^N^	*N*
S3S	2.60	71	38.39	2.231	0.1455	0.0002	234	141	63.6	0.773
S3P	0.99	195.32	39.52	0.5576	0.1259	0.0002	25.8	21.8	1140	0.832
S5S	3.90	35.11	33.65	2.9778	0.4006	0.0002	408	392	0.00406	0.756
S5P	0.99	112.38	28.29	0.1713	0.2609	0.0002	20.9	23	2230	0.833

Surprisingly here the values of electrolyte resistance are more than 30% higher than those calculated for the previous MEAS but even that, values are acceptable, and they cannot explain on their own the significant differences in terms of functioning. Series connection allows for an increase in the total applied voltage while maintaining the current. This current density is reduced in the 5-cell stack compared to the 3-cell stack, due to the inclusion of MEAs with poorer performance characteristics in the former. Conversely, parallel connection maintains the maximum voltage that the stack can provide but significantly increases the current density, reaching a maximum value of 195.32 mA·cm^–2^ (21.09 A) in the 3-cell stack. The 5-cell stack exhibits an unusual behavior under high current demands, which can be explained by the generation of limitations in the supply of reactants to the electrode surface in the last cells of the 5-cell stack (once 110 mA·cm^–2^ (19.8 A) is exceeded, there are no reactants available to allow normal operation with the current feed rate of 230 mL·min^–1^). It is important to note that these tests study the electrochemical connection, as the feed connection is in series, meaning the output of each cell is connected to the input of the next cell. In both series and parallel connections, the maximum achievable power is similar, around 30.97 mW·cm^–2^ for the 5-cell stack (5.57 W) and 38.95 mW·cm^–2^ (4.21 W) for the 3-cell stack, although the current densities at which the maximums are obtained are very different, being around 35–70 mA·cm^–2^ for the series connection and 100–195 mA·cm^–2^ for the parallel connection. Regarding the results shown by the impedance spectra, it is very important to highlight the increase in electrolyte resistance offered by the series connection compared to the parallel connection, which intensifies with the number of electrically connected cells.

These results also provide an initial basis for assessing the scalability of the modular configuration. The proposed design is inherently scalable from a structural perspective because each fuel-cell frame acts as a repeating module. Nevertheless, increasing the number of cells will require optimization of reactant distribution, pressure-drop management, water handling, thermal management, and electrical interconnection. These aspects become progressively more important as stack size increases, as suggested by the reactant limitations observed in the five-cell configuration. Integration into agricultural energy-management systems could be achieved using hydrogen storage and regulation equipment compatible with decentralized renewable-energy installations.

### 3.5 Operation Performance Tests

To assess the feasibility of this technology for robust applications in hydrogen storage for agricultural irrigation or hybrid engines in automotive industry (within the framework of broader projects aimed at providing a holistic solution to this challenge) the use of pure oxygen as an oxidant was deemed economically unviable. Therefore, air was selected as the oxidant. It was essential to gather relevant data on the impact of the oxidant on system performance. Additionally, the influence of the fuel flow rate was investigated. As shown in Figure 5, a three-cell stack was previously operated with a hydrogen flow rate of 230 mL·min^–1^. In this study, the system was operated with half that amount (115 mL·min^–1^), using air as the oxidant, to evaluate its performance under more realistic conditions.

For comparison purposes, the stack was first tested under pure oxygen feeding, consistent with the conditions discussed earlier. Thus, Figure 7 shows the polarization curves of each of the three MEAs integrated into the stack, as well as the resulting polarization curves when the three cells are connected in parallel to power a system capable of delivering more than 0.5 W under galvanostatic operating conditions, when the system is directly fed with oxygen. The figure also presents results that demonstrate the feasibility of operation, and the power output achieved when operating potentiostatically at 0.15, 0.30, 0.45, 0.60, 0.75, and 0.90 V. When comparing the results shown with those in Figure 5, the limitation due to fuel access becomes evident: when the hydrogen flow rate is doubled (still using pure oxygen), the power output increases by nearly the same proportion (1.59 vs 3.91 W).

**Figure 7:**
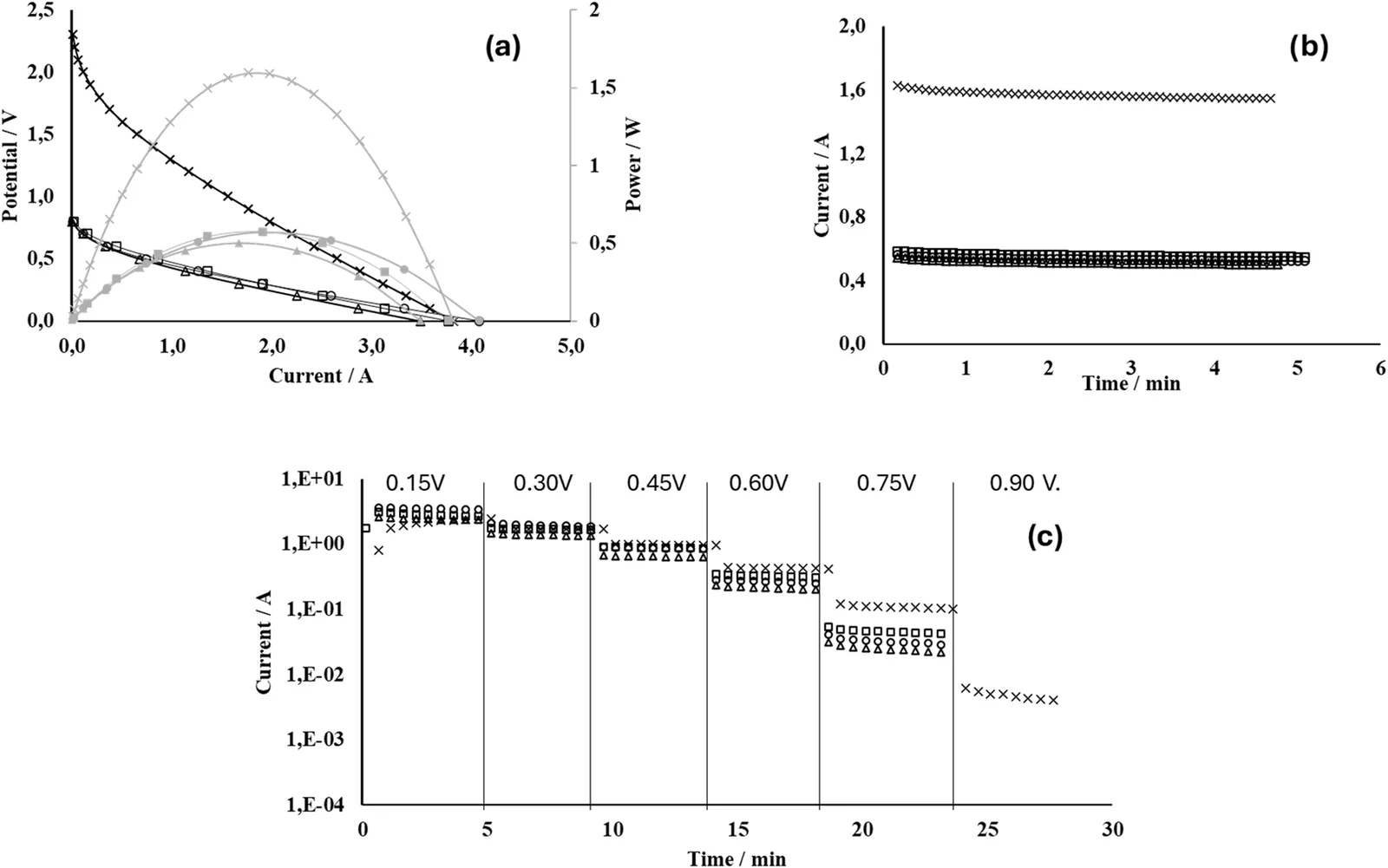
Characterization of the S3P using oxygen as comburent and feeding 115 mL·H_2_ min^–1^. Part (a): Polarization (black symbols) and power (gray symbols) comparative between three single fuel cells and its series connection. Part (b): Changes in voltage during short-term operation tests at 0.5 A. Part (c): Current evolution at various voltages demanded to the cells and the stack. FC1 (□), FC2(○), FC3 (△); S3S (x).

Figure 8 presents the same set of graphs under air-feeding conditions. As expected, performance under pure oxygen is significantly superior, indicating that the device’s operation is limited by the oxidant. The maximum power output of the stack when fed with air is 0.95 W at 1.355 A, which is notably lower than the 1.594 W achieved at 1.769 A under oxygen feeding, highlighting the substantial influence of the oxidant on system performance.

**Figure 8:**
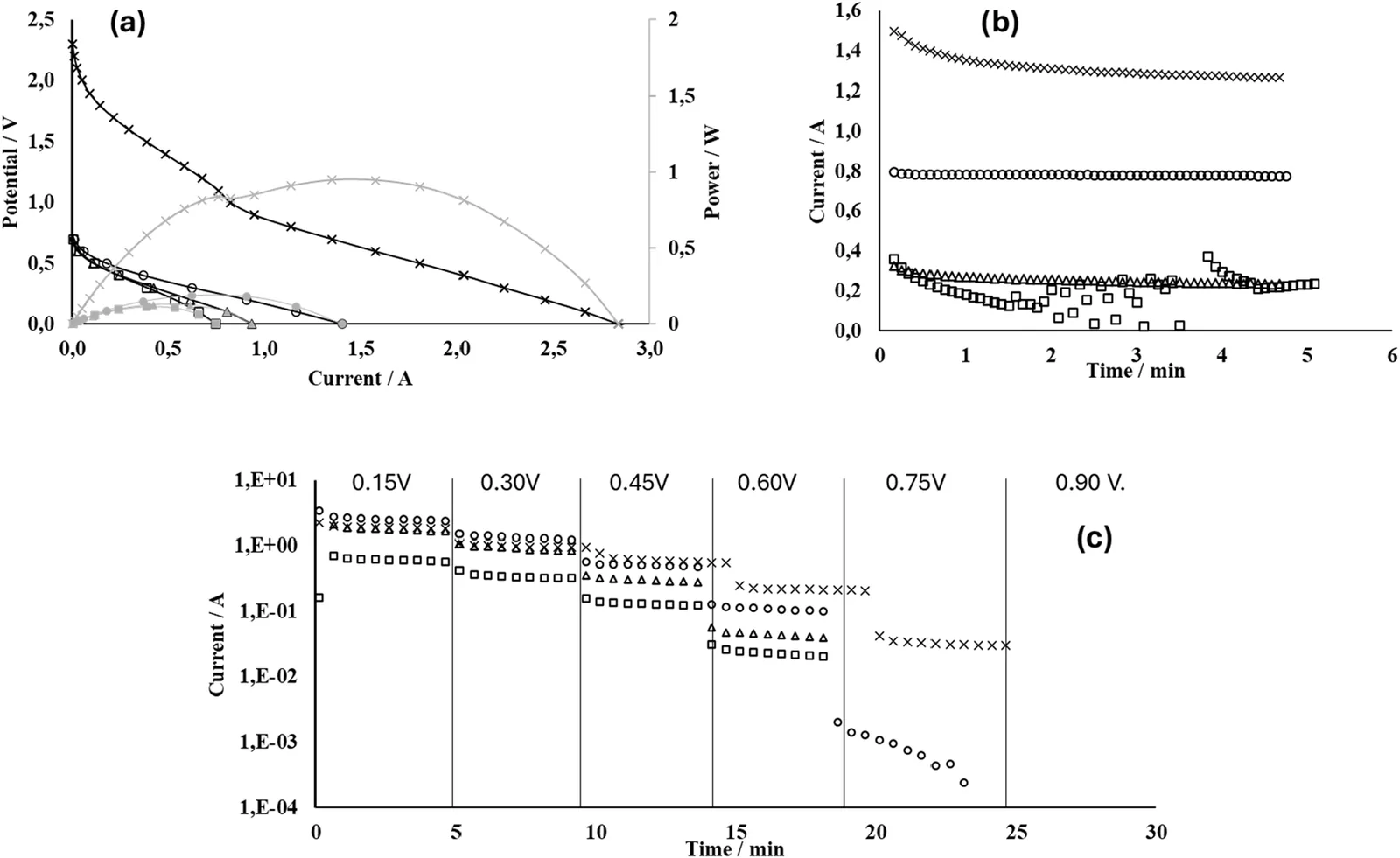
Characterization of the S3P using air as comburent and feeding 115 mL·H_2_ min^–1^. Part (a): Polarization (black symbols) and power (gray symbols) comparative between three single fuel cells and its series connection. Part (b): Changes in voltage during short-term operation tests at 0.5 A. Part (c): Current evolution at various voltages demanded to the cells and the stack. FC1 (□), FC2(○), FC3 (△); S3S (x).

A comparison of the polarization curves for the individual MEAs confirms that this difference stems from reactant limitations, specifically the oxidant. As shown in part (b), even when air is used as the oxidant, the system can still meet the desired specifications; however, the performance variability is greater than in the oxygen-fed configuration.

Similar to the oxygen-feeding tests, the figure also includes results demonstrating the feasibility of operation under potentiostatic control at 0.15, 0.30, 0.45, 0.60, 0.75, and 0.90 V. These results clearly show the reduced performance under air feeding, with the system unable to reach the highest target cell voltage.

Thus, the comparison of the polarization curves of the individual cells within the stack clarifies that the oxidant becomes the limiting factor earlier, with maximum current intensities around 1 A in the latter case, almost four times lower than those achievable when the system is supplied with air, and more than ten times lower than when a higher hydrogen flow rate is also provided. As remarked in part (b) of the Figures 7 and 8, where the cell is operated at different voltages, air feeding reveals greater discrepancies in performance compared to oxygen, highlighting the limitations more clearly. Except at the highest voltage, the cell maintains nearly constant current output, ensuring stable operation.

These conditions were used to evaluate the operational performance of the S3S stack under discontinuous operation, in which this device was directly fed with air and hydrogen stored in five 1-L canisters pressurized at 4 bar, until depletion and subsequent immediate replacement by the next canister. This setup emulates the expected scenario in a hydrogen-based energy regulation system, where the goal is to maintain a continuous electrical supply of 0.5 A to a device (simulated here using a potentiostat).

Figure 9 illustrates the evolution of the energy output from the stack over a 3.29-h period during which five hydrogen canisters were sequentially depleted. Throughout this operation, a total electric charge of 1.65 Ah was consumed, resulting in the delivery of 1 Wh of energy. The figure also displays the cell voltage, current, and power, along with the hydrogen consumption from each canister.

**Figure 9:**
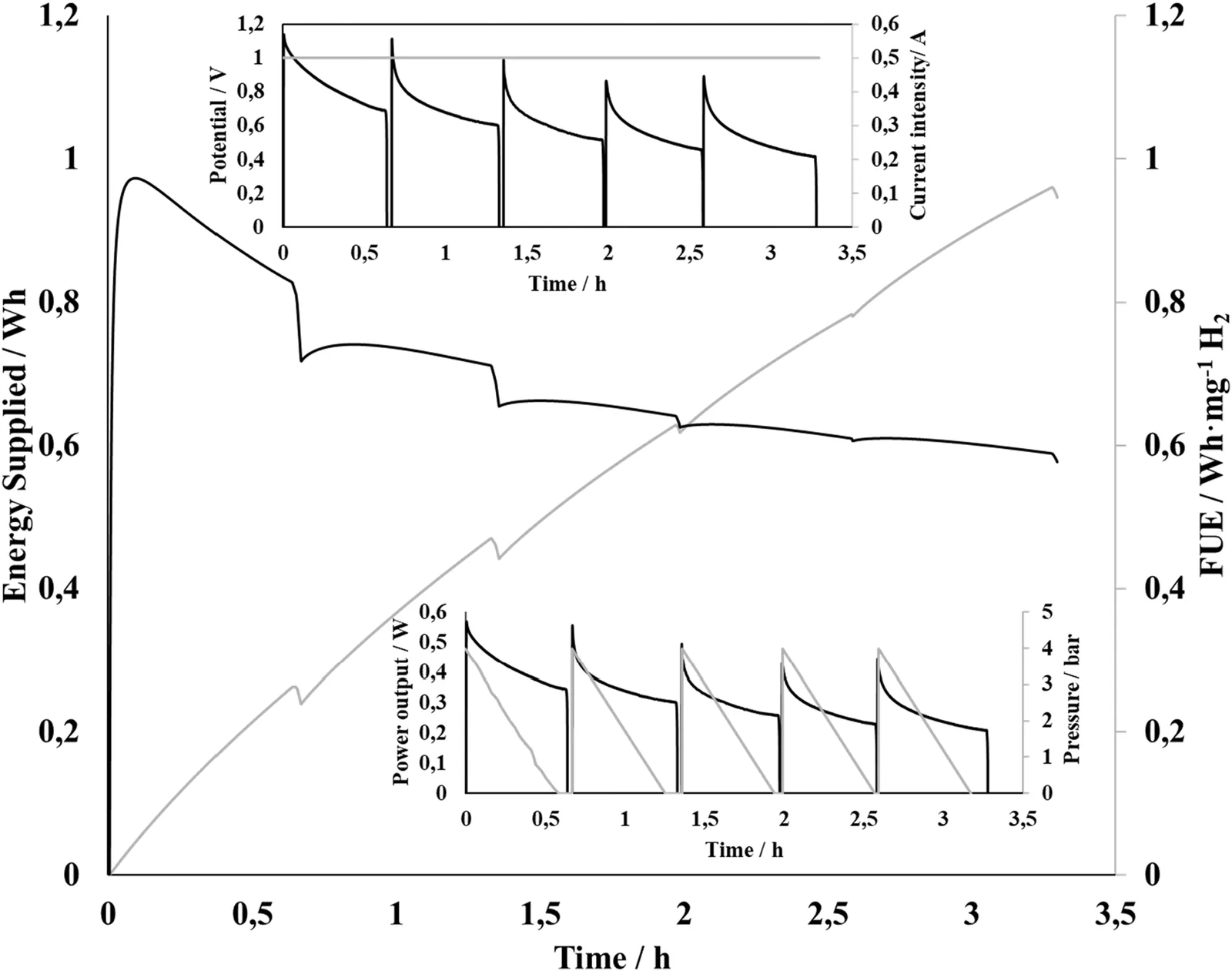
Total energy delivered/supplied (black line) and fuel utilization efficiency (gray line) during the operation of stack S3S, sequentially fed by five hydrogen canisters pressurized at 4 bar until depletion and replacement by the next one. Upper inset: variations in voltage and current overtime. Lower inset: variations in pressure (gray line) and output power (black line) over time.

As observed, each canister is depleted in just over 30 min. In each cycle, the power output follows a decreasing exponential trend, contrasting with the nearly linear pressure drop—consistent with constant flow operation. A clear trend is evident in the ratio between the energy generated and the hydrogen consumed: while the total energy output increases with each additional canister, the efficiency (quantified as hydrogen utilization efficiency in Wh·mg^–1^ H_2_) remains constant within each cycle.

As hydrogen from successive canisters is introduced, the energy generated per unit mass of fuel gradually decreases, but this trend stabilizes over time. This indicates that the system reaches a steady-state efficiency after approximately 1.5 h of operation. Based on the total energy delivered and the amount of hydrogen consumed, the fuel utilization efficiency of the stack is estimated at 0.6 Wh·mg^–1^ H_2_, which is considered acceptable under the realistic operating conditions employed in this study.

These results confirm the feasibility of using 3D-printed frames to assemble fuel cell stacks suitable for robust operation. This approach enables easy replacement of damaged components during operation, reducing dependence on external suppliers.

Nevertheless, the duration of this proof-of-concept test does not allow conclusions to be drawn regarding the long-term durability of the system. Although a long-term durability study was beyond the scope of the present proof-of-concept work, the most relevant degradation mechanisms are expected to be associated with conventional PEMFC components, including MEA degradation, catalyst aging, and membrane dehydration or flooding, rather than with the printed frame itself. The modular design facilitates preventive and corrective maintenance by enabling the rapid replacement of individual MEAs or sealing components. Future studies will therefore focus on accelerated aging and cycling tests under realistic operating conditions.

Thus, although the performance results obtained still offer considerable room for improvement, they clearly demonstrate the viability of the initially defined objectives and the potential for integration into energy regulation systems. Such systems are particularly relevant for applications like efficient irrigation in regions where water scarcity necessitates nighttime water delivery to minimize evaporation losses and as well in the automotive industry where there is still slot for the development of new technologies that can benefit from the use of renewable energy. However, these potential environmental benefits should be quantified through a full life-cycle assessment once sufficient data on material consumption, component lifespan, replacement frequency, and long-term operation become available.

In both contexts, renewable energy storage becomes essential. Conventional technologies, such as lead-acid batteries, advanced lithium-ion systems, or even redox flow batteries, present significant limitations that justify the exploration of alternative solutions like the one proposed in this study.

However, the comparison between fuel-cell and battery-based storage systems should consider their different operating principles and intended applications. Unlike batteries, whose autonomy is determined by the initially stored charge, fuel-cell systems can extend their operating time by replacing or refilling hydrogen canisters without modifying the electrochemical stack. In addition, maintenance can be performed at the component level by replacing individual MEAs or seals. On the other hand, fuel-cell systems require hydrogen production, storage, and supply infrastructure and presently involve greater system complexity than standalone battery solutions. Therefore, the two technologies should be regarded as complementary rather than directly competing energy-storage solutions.

## 4 Conclusions

The following conclusions were obtained from this study:

- 3D-printed resin structures proved to be a viable alternative to traditional graphite bipolar plates, enabling the fabrication of lightweight, low-cost, and easily replaceable fuel cells. These structures withstand temperatures up to 80 °C and allow for efficient water management in stacked configurations.
- Thermal and chemical tracer tests revealed favorable flow distribution, short dynamic response times (∼2–2.5 min), and high Peclet numbers, indicating dominant convective transport, an essential feature in fuel cell design.
- Tests with three individual cells, each equipped with different MEAs but fabricated under identical conditions, demonstrated outstanding reproducibility. Optimal current densities of 150–165 mA cm^–2^ (5.4–5.9 A) and peak power densities above 55 mW·cm^–2^ were achieved at 20 °C, using 230 mL·min^–1^ of hydrogen and 200 mL·min^–1^ pure oxygen in the 36 cm^2^ electrodes single cell.
- Series connection of two cells nearly doubled the maximum power output compared to individual cells. However, the inclusion of a lower-performance MEA in a three-cell stack reduced the maximum power density to 30 mW·cm^–2^, highlighting the critical role of MEA quality in overall stack performance.
- Series configuration enabled maximum power outputs of 5.36 W for the stacks of 5 MEAs and 3.91 W for the stack of 3 MEAs, although the current densities at which the maximums are obtained are very different, being around 30–70 mA·cm^–2^ for the series connection and 110–200 mA·cm^–2^ for the parallel connection
- Important influence of the fuel flow rate dosed reaching maximum power of 3.91 W for 230 mL·min^–1^ vs 1.594 W with half the hydrogen flow when the cells were fed with oxygen as oxidant. Although performance with pure oxygen was superior, the system operated stably with air, reaching a maximum power of 0.95 W at 1.355 A. This validates its use in agricultural or automotive applications where operational cost is a key factor.
- Under realistic operating conditions, the S3S stack maintained a constant current of 0.5 A for 3.29 h using sequentially five 1-L hydrogen canisters at 4 bar. Hydrogen utilization efficiency remained stable at 0.6 Wh·mg^–1^ H_2_, reaching steady-state after 1.5 h.
- Compared with battery-based storage, the proposed fuel-cell system offers complementary advantages associated with modularity, replaceable fuel canisters, and component-level maintenance, particularly for decentralized applications requiring extended autonomy.
